# Analysis of oxygen isotopes of inorganic phosphate (δ^18^O_p_) in freshwater: A detailed method description for obtaining oxygen isotopes of inorganic phosphate in environmental water samples

**DOI:** 10.1016/j.mex.2022.101706

**Published:** 2022-04-16

**Authors:** Catharina S. Nisbeth, Federica Tamburini, Jacob Kidmose, Søren Jessen, David W. O'Connell

**Affiliations:** a Department of Geosciences and Natural Resource Management (IGN), University of Copenhagen, Øster Voldgade 10, 1350 Copenhagen K, Denmark; b Institute of Agricultural Sciences, ETH Zurich, 8315 Lindau, Switzerland; c Geological Survey of Denmark and Greenland (GEUS), Øster Voldgade 10, 1350 Copenhagen K, Denmark; d Department of Civil, Structural and Environmental Engineering, Trinity College Dublin, College Green, Museum Building, Dublin 2, Ireland

**Keywords:** Isotope tracing, Phosphorus, Nutrient biogeochemistry, Critical source areas, Eutrophication, Water quality

## Abstract

The ability to identify the origin of phosphorus and understand processes controlling P cycling is essential for designing effective mitigation and restoration of eutrophic freshwater ecosystems. The oxygen isotope composition of orthophosphate (δ^18^O_p_) has significant potential as a tracer for P entering freshwater ecosystems. However, methods of analysis of δ^18^O_p_ are still in their preliminary stages and have proven challenging to implement for new practitioners. In order to achieve progress in developing the application of δ^18^O_p_ signatures as a tracing tool, there is a need to eliminate the methodological challenges involved in accurately determining δ^18^O_p_. This protocol article describes the various steps needed to concentrate and isolate orthophosphate in freshwater samples into an adequately pure Ag_3_PO_4_ analyte, without isotopic alteration during processing. The protocol compiles the disperse experiences from previous studies, combined with our own experience. The twofold aim of the paper is toprovide a baseline for an increasing standardisation of the silver phosphate purification method associated with analysis of the oxygen isotope composition of orthophosphate (δ^18^O_p_), and to foster new research in the applicability of δ^18^O_p_ signatures for P source tracing in catchment science.

## Graphical abstract

**Figure:**
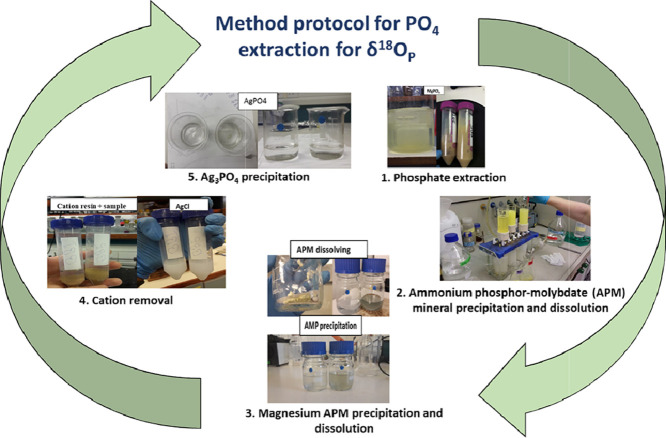

Specifications table

**Table:** 

Subject Area:	Environmental Science Environmental Science Environmental Science
More specific subject area:	*Stable Isotope Tracing*
Protocol name:	Oxygen isotopes of inorganic phosphate (δ^18^O_p_) in freshwater
Reagents/tools:	*Not applicable – listed in document*
Experimental design:	Methods for analysis of δ^18^O_p_ are still under development and have various challenges associated with them for new practitioners. This protocol article outlines and describes the various sequential steps required to concentrate and isolate orthophosphate in freshwater samples without oxygen isotopic alteration into the form of a pure Ag_3_PO_4_ analyte. The protocol compiles the experiences from previous studies combined with our own experience. The two major aims of the paper include the provision of a baseline for an increasing standardisation of the δ^18^O_p_ method, and to foster new research in the applicability of δ^18^O_p_ signatures for PO_4_^3−^ tracing and cycling in the environment.
Trial registration:	*No applicable*
Ethics:	*Not applicable*
Value of the Protocol:	• *Powerful tracer technique to indicate earth systems processes which impact P cycling in the environment.* • *Significant P source tracing technique for both urban and rural aqueous environments.* • *Complimentary technique to use concurrently with other stable isotope tracers, such as stable isotopes of oxygen and hydrogen in water.*

## Introduction

Eutrophication of freshwater ecosystems is often controlled by the availability of phosphorus (P) [2,^12^,37]. Identifying the various potential phosphorus sources and understanding the processes controlling P cycling within freshwater ecosystems is essential for the restoration and management of eutrophic aquatic ecosystems [7,^24^,29]. This is not a trivial matter and requires an appropriate tracer [14].

Organic and inorganic P forms are primarily bonded strongly to oxygen (O), which has three stable isotopes. Accordingly, the oxygen isotope of inorganic phosphate (δ^18^O_p_) has been suggested as a significant prospective tool to trace sources of P and probe P cycling and transformations [1], [2], [3],^13^,23].

The P-O bond in orthophosphate is resistant to inorganic hydrolysis at temperatures and pH levels found in natural abiotic aquatic ecosystems [1,^21^,22]. Subsequently, the δ^18^O_p_ value in abiotic aquatic ecosystems will reflect the isotopic signature of the P sources [32,40].

Biological mediation and hydrolysis of organic compounds containing P may be extremely important parts of the P cycle within certain freshwater ecosystems, which results in an alteration of the source δ^18^O_p_ signatures. Consequently, the δ^18^O_p_ value in aquatic ecosystems will only effectively reflect the isotopic signature of the PO_4_^3^^−^ (hereafter referred to as P*_i_*) sources when the biological activity is relatively low compared to the input of orthophosphate which often occurs during periods of relatively low temperature. The various isotopic alteration processes are, however, associated with distinct isotopic fractionation effects. The δ^18^O_p_ signal can therefore also be utilized to obtain insight into the degree of P cycling and identify the ongoing P alteration processes.

### The δ^18^O_p_-method via Ag_3_PO_4_ precipitation

The most popular method for determination of δ^18^O_p_ is by thermal conversion/elemental analyzer isotope ratio mass spectrometry (TC/EA-IRMS) of a P*_i_* precipitate, generally in the form of silver(I) phosphate (Ag_3_PO_4_) [7,^9^,^10^,^23^,^30^,^32^,40]. TC/EA-IRMS has many advantages over the traditional fluorination technique in that (*i*) small PO_4_ quantities are required for the analysis (yielding ∼300–600 *µg* Ag_3_PO_4_); (*ii*) dangerous chemicals are avoided, such as BrF_5_, F_2_ or ClF_3_; and (*iii*) measurements are automated [36]. The Ag_3_PO_4_ precipitate is preferred as it is less hygroscopic than e.g. bismuth(III)-phosphate (BiPO_4_), is stable, has low solubility, and results in better O yield during quantitative conversion of the PO_4_-O to CO-O, and requires less preparation time [4,8].

### Approaching a uniform P_i_ extraction method via Ag_3_PO_4_ precipitation

Several detailed protocols for the extraction of P*_i_* via precipitation of Ag_3_PO_4_ from different matrix solutions such as fresh and ocean waters and soil extractions exist [3,^9^,^11^,^23^,^32^,40]. The major techniques for these protocols have been summarized by Paytan & McLaughlin [29] and Davies et al. [5].

For water samples, the broadly common sequence of steps for Ag_3_PO_4_ precipitation is this: (*i*) P*_i_* is quantitatively removed from the sample through magnesium-induced co-precipitation (MagIC) by brucite [16]; (*ii*) redissolution of the brucite-pallet in an acid matrix, which resuspends the P*_i_* in the solution; (*iii*) removal of other interfering sources of O, such as dissolved organic matter (DOM), by using anion exchange resins and/or sequential precipitations; (*iv*) removal of potentially interfering cations using a cation exchange resin; (*v*) precipitation of Ag_3_PO_4_. All steps are designed to inhibit isotopic fractionation.

Despite the several existing protocols and the review papers by Paytan & McLaughlin [29] and Davies et al. [5] focusing on analysis of the δ^18^O_p_ of inorganic phosphate, and despite numerous articles describing δ^18^O_p_ application in different aquatic environments, there currently exists no comprehensive protocol for precipitation of Ag_3_PO_4_ for freshwater matrices. In addition, some of the common steps were originally developed and documented for other conditions than they are now applied on. For example, the MagIC steps’ quantitative P*_i_* removal was well documented, but for the matrix of oceanic seawater, which is relatively invariable compared to freshwater matrices. Nevertheless, it has nearly entirely been applied to freshwater samples.

Furthermore, due to the complexity of the method it has been proven challenging to implement for new practitioners. This is a notable problem as the applicability of the δ^18^O_p_ method is contingent upon an extended knowledge of the δ^18^O_p_ signatures and understanding of the systematics of the various isotopic alteration processes [29,38].

Hence, to make the method as widely and practically applicable as possible, especially for researchers who are new to the field, and to facilitate a baseline for a coherent future method development aiming at freshwater systems, there is a need for a detailed method description for the Ag_3_PO_4_ precipitation method. The present technical note aims to address the needs by (*i*) describing each step of the Ag_3_PO_4_ precipitation method in detail; (*ii*) explaining the historical background and reasoning behind each step; (*iii*) compiling from the literature the (lacking) documentation of individual steps; and (*iv*) giving practical advice and suggestions to tackle potential challenges which may arise when applying the method, as it is, under different scenarios.

## Protocol for freshwater δ^18^O_p_ determination

### Reading guide for the protocol

The protocol is for the concentration and isolation of P*_i_* from freshwater samples and results in an adequately pure solid silver phosphate crystal (Ag_3_PO_4_), without isotopic alteration. The subsequent TC/EA-IRMS analysis of the δ^18^O_p_ determination is not described. For the latter, we refer to Tamburini et al. [32] or Davies et al. [5].

The protocol can be used when water sampling volumes are not restricted. In situations where sampling is difficult and sample volumes limited, we refer to the method presented by Goldhammer et al. [9]. If the goal is to determine δ^18^O_p_ from a sediment sample, we refer to the P extraction method presented by Tamburini et al. [32].

The protocol is presented in the following three sections: Section 2.2 *‘Freshwater sampling’,* Section 2.3 *‘Quantitative P_i_ removal by the MagIC method’*, and Section 2.4 *‘Purification and silver phosphate precipitation’*.

Each section is divided into main steps presented by roman numerals and each main step is further subdivided into substeps indicated by letters from the Latin alphabet.

Three different remarks will be presented throughout the protocol:

**Table:** 

*Notes*……	Specific concerns to be aware of when performing one of the substeps.
*This study experienced*……	Phenomena this study has experienced that have not been presented in earlier method descriptions.
*Differences between various*	
*protocols exist……*	Draws attention to steps where there are inconsistencies between already published δ^18^O_p_ methods.

The protocol compiles the disperse experiences from previous studies, combined with the experience associated with this study. Description of the preparation of all used chemicals and reagents are provided in Appendix A.

### Freshwater sampling

The amount of water to be sampled depends on the P*_i_* concentration of the sampled water itself. It is recommended to sample a minimum of 20 *µmol* of P*_i_*. This will provide enough P*_i_* to allow some losses from one step to the next and thus an easier handling of the protocol. This can lead to required water volumes of more than 200 *L* as the P*_i_* concentrations often are less than 0.4 *µM* in freshwater ecosystems, unless significantly affected by human activity or high natural sources of P [17,28]. In situations where the required water sample volumes exceeds 1000 *L* we recommend the method presented by Tcaci et al. [34].

It is important to take the necessary precautions in relation to the type of water being sampled. This is especially true when sampling anoxic and Fe^2+^-rich water were P*_i_* co-precipitation with Fe(III)-(hydr)oxides (henceforth collectively referred to as Fe-oxides), forming upon contact which atmospheric O_2_, could immediately occur [31]. Thus, different sampling approaches are needed when working with either oxic or anoxic samples:

**Step I: Freshwater sampling**

A freshwater sample can be obtained in the following way:

(*a*) Prior to sampling, acid-wash, rinse with deionized distilled water (DD-H_2_O), and air dry a high density polyethylene (HDPE) collection container. If planning on sampling anoxic and Fe^2+^-rich water, additionally flush the container with N_2_ gas and seal the container. (*b*) At the sampling site, fix a piece of nylon mesh on the opening of the collection container (oxic water sampling) or attach the nylon mesh to the tip of the sampling tube, submerged in the collection container (ferrous water sampling) to filter out coarser material. The mesh size depends on practicalities; decide on a size range which allows a decent flow of water without clogging. A 10 µm nylon mesh for lake, stream and groundwater was used, collecting about 1 L per minute using a peristaltic pump. (*c*) Rinse the polyethylene container three times with sampling water before final filling. For ferrous water sampling rinse and fill the container by pumping water through the submerged tube into the container and let the water overflow for an extended period of time. At the final filling, prevent an air headspace in the container before closing it. (*d*) Collect a parallel water sample (minimum 10 *mL*) for measurement of P*_i_* concentration and δ^18^O of water, i.e. δ^18^O_w_.

#### Introducing an optional extra filtration step prior to the MagIC method

Dissolution of particulate-bound P*_i_* and/or acid hydrolysis of particulate organic P may occur after water sampling. Accordingly, a standardized filtration protocol prior to the MagIC method is potentially required. So far, there is no clear guideline regarding adequate filtration requirement for freshwater samples. If necessary particulate organic matter has typically been removed from freshwater samples by filtration through a 0.45 µm GF/F filter [5,^7^,19]. Alternatively, one could use a sequence of 1 µm and 0.2 µm polypropylene cartridge filters under low pressure. For ferrous water samples, however, a lengthy filtration procedure (slow pumping velocity) could alter the water sampling, depending on the effect of co-precipitation of P*_i_* with iron oxides. Clearly, the magnitude of the error introduced by allowing particulates into high-volume samples requires attention in future research. When working with freshwater samples, filtration of the HNO_3_ solution after dissolution of the last MagIC pellet (Step VI) could be an alternative solution to the extra filtration step. Nevertheless, to our knowledge, this still needs to be elucidated further.

### Quantitative P_*i*_ removal by the MagIC method (duration: 1 day)

The MagIC technique concentrates and ideally isolates P*_i_* from the majority of other dissolved ions, dissolved organic P (DOP) and DOM in the water sample [16,35]. Consequently, the method enabling a more manageable P*_i_* sample size for further treatment prior to the purification and final Ag_3_PO_4_ precipitation steps.

**Step II: Magnesium-induced co-precipitation of dissolved P*_i_* (MagIC)**

**Fig. 1:**
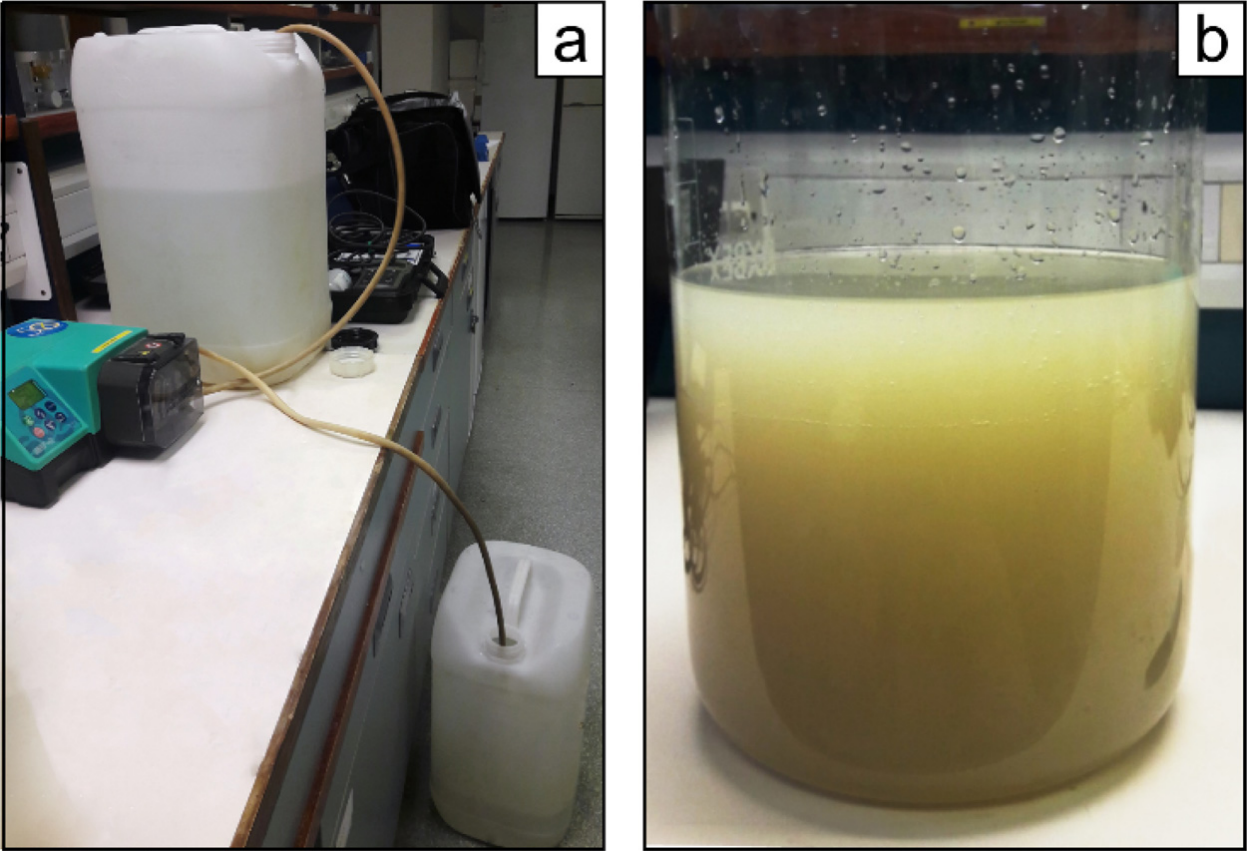
(a) Removing the supernatant from the brucite flocs by siphoning, using a peristaltic pump. (b) Brucite flocs transferred to a beaker after discarding the supernatant.

Magnesium-induced co-precipitation can quantitatively remove dissolved P*_i_* by adsorption onto Mg(OH)_2_ (brucite), initiated by addition of NaOH which raises the pH. Brucite can precipitate at any temperature, but temperatures should be kept low (5–10*°C*) in order to keep microbial activity at a minimum. Furthermore, the water samples need to be processed immediately after sampling to avoid potential microbial alterations. The procedure of the brucite precipitation step is as follows:

(*a*) Discard some of the sampled water to ensure space for the reactants in the polyethylene container. (*b*) Add 3 *M* MgCl_2_ until the solution achieves a final concentration of ∼55 *mM* Mg^2+^ corresponding to the Mg^2+^ concentration found in seawater [16,35]; for example, this corresponds to the addition of 1 *L* of 3 *M* MgCl_2_ to 50 *L* freshwater sample. Mix well. (*c*) Then add 1 *M* NaOH equivalent to 0.5*%* of the sample solution volume [35] and mix again. Check with pH indicator strips that the pH becomes between 9 and 10, as alkaline conditions facilitate brucite precipitation better than acidic conditions [35]. If pH <9 add more 1 *M* NaOH and mix simultaneously. (*d*) Allow the brucite flocs to settle by gravity. Check continuously that the pH is >9 in order to prevent the brucite (and co-precipitated phosphate) from dissolving into solution. (*e*) Then remove the supernatant. With large sample volumes, this can be done by siphoning or using e.g. a peristaltic pump (Fig. 1a). The brucite flocs left after removing the supernatant might make up several liters of sludge (Fig. 1b). (*f*) Check the absence of P*_i_* in the supernatant, e.g. by using the spectrophotometric molybdate blue-method [26]. Discard the supernatant if P*_i_* has been 100% stripped from the sample solution. If P*_i_* is still present, add additional NaOH to the supernatant and combine all the precipitated brucite.

*Notes* (*i*) Excess of NaOH does not improve P_i_ co-precipitation removal as the resulting higher pH decreases PO_4_ adsorption. Rather, excess NaOH has the drawback that it yields a larger mass of brucite flocs which subsequently must be dissolved in a larger volume of acid [16]. (*ii*) If the suspension is left for longer than it takes the brucite flocs to settle P*_i_* may start to desorb from the brucite flocs, probably because recrystallization of the brucite lowers the surface area [3]. This can be prevented by maintaining the solution pH at 9–10 by further NaOH addition.

*Differences between various protocols exist* regarding the precipitation approach of brucite exists: Joshi et al. [15] initially prepared a concentrated MagIC colloidal solution in a split of the sample solution (200-300 *mL*) and concurrently adjusted the pH of the remaining sample solution. They then subsequently mixed the two solutions. The entire volume was then gently shaken continuously to maintain a homogeneous dispersion of colloids and thus maximize the trapping of P*_i_*. Joshi et al. [15] state that this procedure is especially prudent when working with low P*_i_* concentrations. This method procedure has successfully been followed by Yuan et al. [39]. Whether there are discrepancies in the results if one follows this approach instead of the magnesium-induced approach described in Step II above is currently undocumented.

**Step III. Sample centrifugation**

The brucite flocs can be separated from the solution by centrifugation. Do the following:

(*a*) After completing Step II, immediately centrifuge the collected brucite floc sludge (Fig. 1b) at 3500 *rpm* for 10 min, and discard the supernatant. Timewise, it is recommended to use as large centrifuge tubes as possible, e.g. 250 *mL* tubes. The supernatant should appear clear after the centrifugation.

*Differences between various protocols exist* regarding the recommended centrifugation rotation speed: Karl & Tien [16] recommend a low speed (1000 rpm for 1 *h*) as high g-forces (experienced at >12000 *rpm*; [16]) make the settled brucite flocs harder to dissolve subsequently and do not improve the separation from the supernatant. In contrast, Goldhammer et al. [9] recommends a high rotation speed (10000 *rpm* for 15 minutes) to ensure complete settling of the fine crystalline Mg(OH)_2_, which have a significantly different δ^18^O_p_ signature than the δ^18^O_p_ of coarser brucite flocs. Nevertheless, we followed McLaughlin et al. [23]’s compromise were a rotational speed of 3500 *rpm* for 10 min was used. This approach was successful followed by Young et al. [38] and Elsbury et al. [7], both working with freshwater samples. An alternative to centrifugation is gravitational separation used by Colman [3].

**Step IV. Brucite dissolution**

The co-precipitated P*_i_* is re-liberated by dissolving the brucite flocs in 1 *M* HNO_3_. The technique is as follows:

(*a*) Add 1 M HNO_3_ to the centrifuge tubes used for Step III. The required added volume depends on the quantity of brucite flocs. Add until the brucite can be easily removed from the centrifuge tubes. Be sure to use the minimum amount of acid to minimize acid hydrolysis [16]. (*b*) Combine the dissolved brucite flocs from the centrifuge tubes. (*c*) Adjust the final pH to ca. 1 using 1 *M* HNO_3_ (use indicator pH test strips), as brucite is first fully dissolved under these conditions; at this point the solution will be liquid and not viscous.

*Differences between various protocols* exist regarding the final pH of the dissolved brucite solution. Colman [3], Goldhammer et al. [9] and McLaughlin et al. [23] all recommend carefully buffering the solution back up to a pH between 4 and 6 after re-dissolution of the brucite is complete, making H_2_PO_4_^−^ the main P*_i_* species in the solution. However, the subsequent purification steps in these three studies (precipitation of cerium phosphate [23] and a pump-based anion-exchange chromatography setup [3,9]) all utilizes a pH of around 6. In the present protocol the subsequent purification steps utilizes the low pH (see Step VII). Adjustment of pH is therefore not applied in the MagIC protocol presented here.

**Step V. Additional MagIC step**

**Fig. 2:**
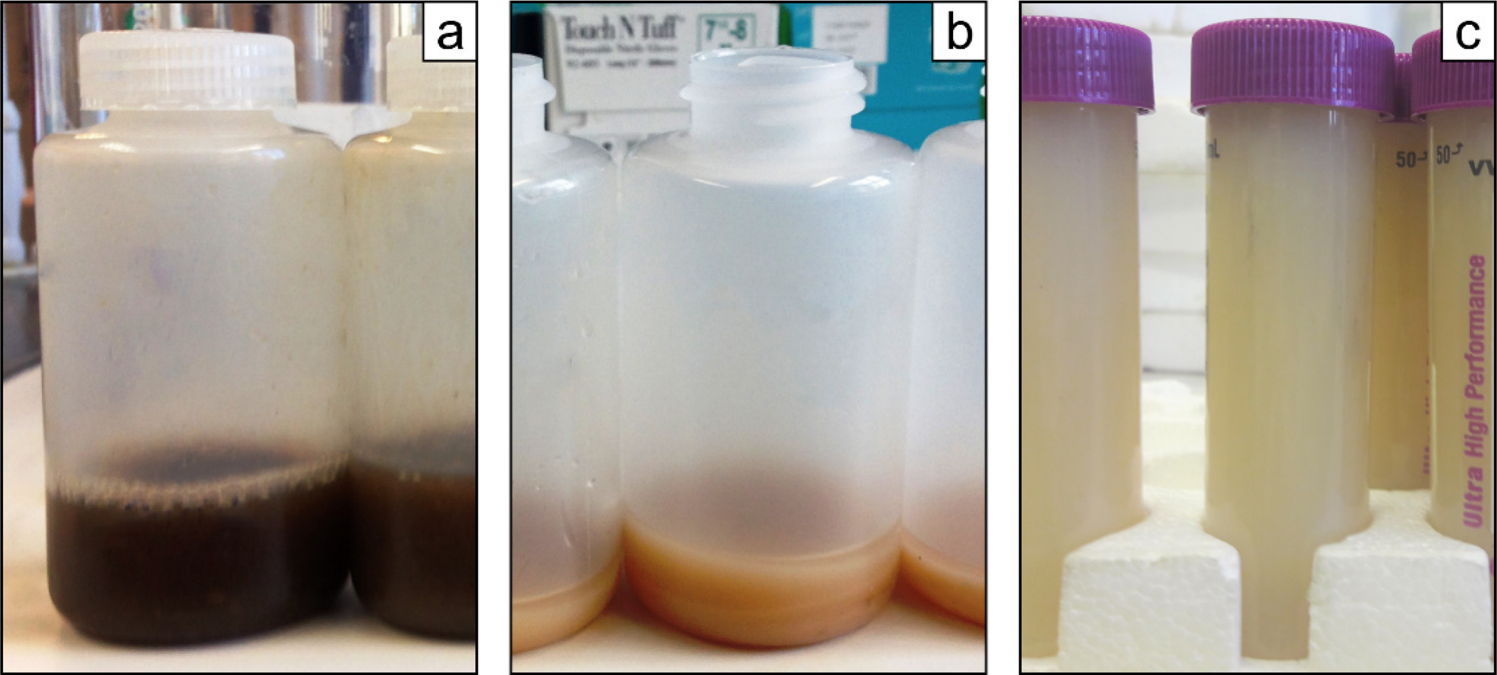
Brucite discoloration of sample with high dissolved organic matter (DOM) content. DOM-rich brucite flocs after (a) the first precipitation, (b) after three HNO_3_ dissolution and NaOH precipitation repetitions and (c) purified brucite (Step V).

If the sample contain organic material, the color of the precipitated brucite flocs become tan or even brown [9,40] (Fig. 2a & b), whereas it should be milky whitish if purified (Fig. 2c) [16]. An additional MagIC step, Step V, is thus required leading to (*i*) further purification of P*_i_* from a matrix with potential contaminants and (*ii*) higher concentrated P*_i_* brucite flocs [3,9]. Step V proceeds as follows

(*a*) Raise the pH of the dissolved brucite to about 10–11 by adding 1 *M* NaOH (do not add the 3 *M* MgCl_2_ solution). Brucite precipitation occurs at pH 9. (*b*) Then, repeat Step III and Step IV. A final pH of 1 is still required. (*c*) Repeat Step V until discoloration disappears; up to five repetitions may be necessary [9].

*Note* Samples with a brownish color have also been successfully purified by using a column system presented by Colman [3].

*This study experienced* that even if the brucite flocs are not discolored, it is recommended to conduct Step V once, as it gave a significantly higher success rate regarding the final Ag_3_PO_4_ precipitation.

**Step VI. Filtration**

After completing Step V one should be left with a solution with a pH of ∼1. There might still be some undissolved particles left, in some cases with a brownish color. The final step of the MagIC protocol separates particles insoluble under acid conditions from the acid dissolve brucite flocs, by vacuum filtration. Do the following:

(*a*) Filter the dissolved brucite using a 0.7 µm GF/F filter. It may be necessary to centrifuge first if the floc is not fully dissolved in acid at pH 1.

*Note* This step is especially important if the dissolved brucite remains brownish after repeating step V a couple of times.

After Step VI, it is recommend to immediately proceed with the subsequent purification step (Step VII). If the samples needs to be stored and/or transported then do not dissolve the brucite flocs after the last brucite precipitation, and store the sample in the fridge. The brucite flocs should be dissolved in acid (Step IV) only just before the first purification step (Step VII).

#### Evaluation of the MagIC protocol

According to Colman [3] phosphate losses associated with the concentration steps of the MagIC protocol might amount to not more than 5%, estimated from sea water samples. This is conformity with preliminary data from this study.

The MagIC protocol was initially developed for samples of seawater [16] which has a nearly constant matrix composition independent of the sampling site. In addition, seawater P*_i_* concentrations are rarely high enough to challenge the quantitative P*_i_* removal in Step II. In contrast to seawater, when working with freshwater samples, the matrix can vary significantly and the concentration of DOM is often much higher. Both of these conditions can potentially inhibit the feasibility and accuracy of the MagIC method when working with freshwater samples.

Recent research has demonstrated that organic P compounds and oxyanions including nitrate, sulfate and HCO_3_^−^ can also co-precipitate with the brucite, as they have a similar affinity for brucite as P*_i_*. This consequently may result in interference during the final determination of δ^18^O_p_ [15,34]. An extra step prior to the MagIC treatment may thus be required. E.g., HCO_3_^−^ can be removed by acid treatment forming degassing CO_2_ [15]. Additional amendments and additions to the MagIC methodology might be necessary when working with some freshwater samples. This is an important subject, which needs to be investigated in more detail.

The acid dissolution of brucite (i.e. Step IV) can also be a potential challenge when processing organic rich samples. During that step, acid hydrolysis may occur, which may potentially convert organic P into new P*_i_* in which water-O from the ambient environment may be incorporated [25]. The newly generated P*_i_* will potentially be incorporated in the Ag_3_PO_4_ crystals, subsequently altering δ^18^O_p_ signature of the sample. Hydrolysis of a large range of DOP compounds have however been proven to be negligible at extreme pH conditions in the time frame used in routine laboratory processing of samples [3,^13^,^29^,35]. Yet it is important to keep in mind the significant variation of the freshwater matrix, and thus the vast array of organic P compounds which react diversely [3,35]. When processing organic-rich freshwater samples one could use ^18^O-labeled and unlabelled reagents on replicates of the same sample to trace and correlate the impact of acid hydrolysis.

In literature, interfering O-bearing compounds potentially incorporated into the Ag_3_PO_4_ seems to be of bigger concern [5,^9^,^11^,^23^,32] than the probability of acid hydrolysis during brucite dissolution [13,29].

Alternative methodology seeking to avoid these potential sources of error when working with freshwater samples is emerging. Neidhardt et al. [27] successfully quantitatively removed P*_i_* from freshwater samples by co-precipitation with Fe-oxides. Tcaci et al. [34] present a method favourable when working with freshwater systems with low P concentrations which require very large quantities of water sample (>1000 L). Their methodology involves an initial treatment of the freshwater sample using anion exchange resin to isolate phosphate from contaminant sources of oxygen, whether in organic matter or in the form of other oxyanions.

Based on experience it is not yet possible to conclude that no co-precipitation or acid hydrolysis of organic P compounds occurs in freshwater samples when using the MagIC methodology. Notwithstanding, the MagIC method has the potential to be applied to freshwater samples. The complexity and variation in different freshwater matrix influence the usability of the different methods. Consequently, there might not be a universal protocol regarding the initial quantitative removal of P*_i_* from the water sample. More research needs to be conducted in order to clarify advantages and disadvantages of the different methods.

### Purification and silver phosphate precipitation (duration: 5 to 8 days)

The presented phosphate purification protocol consists of sequential precipitation and recrystallization developed with the specific goal of reducing contamination by dissolved organic matter (DOM) [18,^20^,32]. Briefly, P*_i_* is first precipitated as ammonium phospho-molybdate (APM), and then recrystallized as magnesium ammonium phosphate (MAP). This is combined with a subsequent cation resin treatment followed by elimination of chloride. The purification protocol is presented below.

**Step VII. Ammonium phospho-molybdate (APM) precipitation (duration: two days)**

During the first step of the purification protocol, P*_i_* is scavenged from the acidic dissolved brucite solution by precipitation of APM crystals. This enables the separation and removal of ions and contaminants that are soluble at acidified conditions [15]. The APM precipitation procedure is as follows:

(*a*) Initially, transfer the sample solution (i.e. the dissolved brucite) to an Erlenmeyer flask of suitable volume (sample and reactants' combined volume) and place the flask in a 50 *°C* warm water bath shaker or on a magnetic stirrer with heating set to 50 *°C*. (*b*) If the solution is taken directly from the refrigerator, wait until the sample is close to room temperature before continuing. (*c*) Then add 25 *mL* 35*%* ammonium nitrate reagent, and then slowly add 40 *mL* of the 10*%* NH_4_-molybdate solution. (*d*) Adjust the final pH to ca. 1 using 1 *M* H_2_SO_4_ (use indicator pH test strips). Normally around 1 *mL* is enough; thereby the volume of the sample is not affected too much. (*e*) Leave the solution in the 50 °C warm water bath and shake gently overnight to ensure complete APM precipitation.

**Fig. 3:**
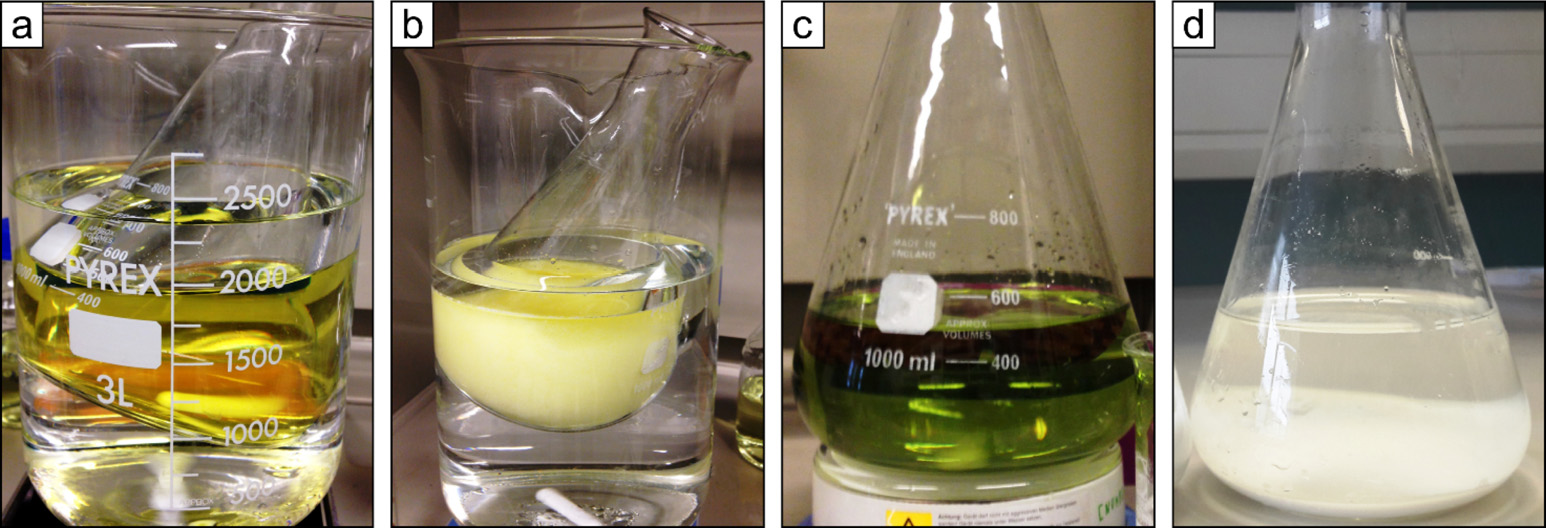
Color of the supernatant and the precipitate in Step VII when (a) optimal precipitation conditions with respect to APM crystals are obtained, (b) APM crystals are forming, (c) alkaline conditions impede APM precipitation and (d) with an unidentified precipitate resulting from incorrect execution of the MagIC protocol.

*Note* If the supernatant turns transparent bright yellow (Fig. 3a) this is an indication that optimal precipitation conditions with respect to APM crystals are obtained. When this color changes to milky yellow, it indicates that APM crystals are forming (Fig. 3b). If no APM crystals have started to precipitate from the heated solution after around 15 min, supersaturated conditions with respect to APM crystals are likely not obtained or pH is not correctly adjusted. First check the pH and adjust if necessary (cf. Step VIId). If still no APM crystals precipitate, add stepwise more 35*%* ammonium nitrate and 10*%* NH_4_-molybdate solution in the 2.5:4-ratio until crystal precipitation is initiated.

*This study experienced* where supernatants were slightly alkaline after Step VIIc, it became bright green (Fig. 3c) and no APM started to precipitate. When adjusting the pH to 1 the supernatant turned to a transparent bright yellow color (Fig. 3a) and APM crystallization immediately began. The slightly alkaline conditions could have affected the dissolution of the brucite flocs, since brucite dissolves at acidic conditions. We also experienced that if the brucite flocs had not been acidified to pH 1 during the dissolution step (Step IV) and/or if the additional MagIC step (Step V) was not conducted, the crystals precipitating in this purification step were white and the supernatant transparent (Fig. 3d). We were not able to accomplish a final precipitation of Ag_3_PO_4_ when we tried to proceed with these white crystals. Accordingly, we suggest that the color of the supernatant and the precipitate can be used as an indicator for (*i*) optimal pH conditions for APM precipitation and (*ii*) whether it is worthwhile to continue. The adjustment of the pH and an introduction of additional MagIC steps were performed simultaneously in the present study. No examination of whether both actions are equally important has been reported nor tested in the present study.

**Step VIII. APM dissolution**

**Fig. 4:**
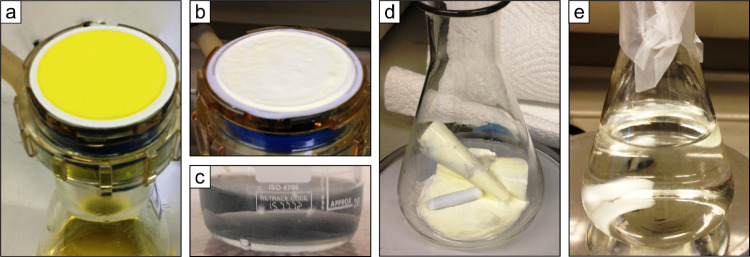
(a) and (b) Two examples of vacuum filtered and 5*%* ammonium nitrate washed ammonium phospho-molybdate crystals (APM) from two different samples. The APM crystals differ in color and size. (c) Greenish discoloration of the dissolved ammonium phospho-molybdate crystals. (d) Ammonium phospho-molybdate crystals (APM) on 0.2 µm cellulose acetate filters, transferred to Erlenmeyer flask for dissolution. (e) Dissolved APM crystal in a NH_4_-citrate solution resulting in a transparent solution.

The P*_i_* is released from the APM crystals by dissolution in an alkaline solution prior to an additional purification step. Conduct the step as follows:

(*a*) Start by separating the yellow APM crystals from the supernatant by vacuum filtration upon a 0.2 µm cellulose acetate filter and discard the supernatant. The filtration time can take several hours and more than one filter may be necessary. Successfully obtained APM crystals from different samples may differ slightly from each other in color and size (Fig. 4a & b). (*b*) Wash the crystals thoroughly with a 5*%* ammonium nitrate solution to rinse off contaminants (>200 *mL*). (*c*) Transfer the filter(s) containing the APM crystals to a 100 *mL* Erlenmeyer flask and place the flask on a magnetic stirrer (Fig. 4d). (*d*) Dissolve the APM crystals in a minimum amount of NH_4_-citrate solution (15–50 *mL*; volume depends on the quantity of formed APM crystals). Start by adding 10 *mL* and then add 5 *mL* aliquots. Work under a chemical fume hood. (*e*) Gently swirl the solution while the crystals are dissolving and wait until the solution becomes transparent (Fig. 4e), which may take up to 15–20 min. Then remove and discard the filter(s).

*Note* Mg^2+^ ions could interfere with dissolution of the APM crystals leading to some crystals not dissolving. In addition, silicates may have formed in the former steps. These are not dissolvable in the NH_4_-citrate solution. Accordingly, some particulate compounds might be left in the solution after it turns transparent. If so, filter again using a 0.2 µm cellulose nitrate filter and discard the filter.

*This study experienced* that the dissolved APM solution at times had a greenish discoloration (Fig. 4c), maybe due to precipitation of silicate molybdate complexes. We tried to continue the protocol with these samples after filtation, which still resulted in Ag_3_PO_4_ crystal precipitation in the last step.

**Fig. 5:**
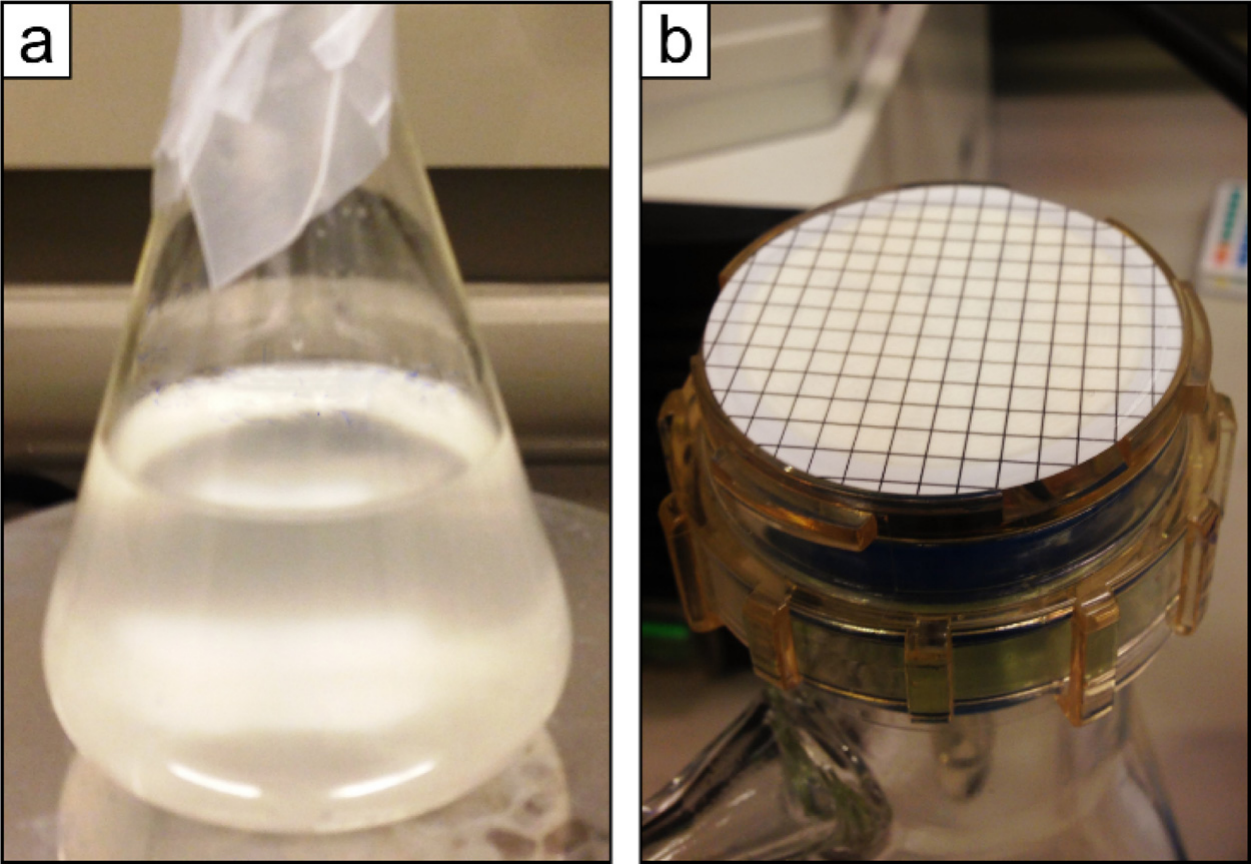
(a) Precipitated magnesium ammonium phosphate crystals. (b) Vacuum filtered and 1:20 ammonia solution washed magnesium ammonium phosphate crystals.

In this step the solution is further purified regarding contaminants and isolation of P*_i_*, by precipitating MAP crystals under alkaline conditions, thus enabling the removal of ions and contaminants that are soluble at alkaline conditions. The MAP precipitation procedure is as follows:

(*a*) Initially add 25 *mL* Mg-reagent to the 100 *mL* Erlenmeyer flask, containing the dissolved APM solution, while stirring. (*b*) Then slowly add about 7 *mL* of the 1:1 ammonia solution. (*c*) Check pH. If pH <8 carefully add more of the 1:1 ammonia solution until the solution acquires pH 8–9 which is the optimum pH for MAP precipitation. MAP crystals should start to precipitate immediately, turning the solution whitish opaque (Fig. 5a). (*d*) Cover the Erlenmeyer flask with parafilm and make mm-size holes for venting. Leave the solution overnight on the magnetic stirrer.

*This study experienced* that it was necessary to add additional Mg-reagent to some of the samples, after adjusting pH to 8–9, in order to achieve supersaturation with respect to the MAP crystals. This was true for the samples were >20 mL of NH_4_-citrate solution had been used to dissolved the APM crystals.

**Step X. MAP dissolution**

P*_i_* is re-released by dissolving the MAP crystals in a minimum amount of HNO_3__:_

(*a*) Separate the white MAP crystals from the supernatant by vacuum filtration upon a 0.2 µm cellulose nitrate filter and discard the supernatant. The MAP crystals are small and may be quite hard to see on the filter by eye, see Fig. 5b. (*b*) Wash the crystals thoroughly with 1:20 ammonia solution (>200 *mL*) to get rid of excess chloride and other contaminants. (*c*) Transfer the filter to a 50 *mL* centrifuge tube (with lid) and dissolve the MAP crystals in a minimum amount of 0.5 *M* HNO_3_ (5–10 mL) by shaking the sample. (*d*) Leave the filter in contact with the acid for at least 15–20 min to ensure that the MAP crystals have dissolved.

*Note* (*i*) It is difficult to assess when the MAP crystals have fully dissolved, since the filters and the crystals are both white. (*ii*) It is extremely important to get rid of excess Cl^−^ ions coming from the Mg solution (i.e. MgCl_2_ and HCl), as more remaining Cl^−^ means that more AgNO_3_ has to be added in Step XII which could entrain the formation of AgO in the final product Step XIII.

**Step XI. Cation removal (duration: three days)**

The presence of cations (primarily Na^+^ and multivalent cations such as Mg^2+^) interferes with the precipitation of Ag_3_PO_4_. Thus a prior cation removal step is a crucial prerequisite for the subsequent successful precipitation of purified Ag_3_PO_4_ [8]. Cations can be scavenged by a proton-charged cation resin, releasing H^+^ to solution, which subsequently reacts with HCO_3_^–^ (if present), forming H_2_O and CO_2_ [3]_._ The purification step is as follows:

(a) Convert a new cation exchange resin AG50WX8 to an H^+^ form by reacting the resin with 7 *M* HNO_3_ overnight on a horizontal shaker. A 7 *M* HNO_3_ volume of 1.5 times the resin volume is recommended. (*b*) The following day, discard the HNO_3_ and rinse the resin thoroughly by mixing it with 1 *L* DD-H_2_O to bring it close to neutrality (pH >5). (*c*) Filter the mixture on a 0.45 µm polycarbonate filter and discard the water. It might take up to several repetitions before a neutral pH is obtained. (*d*) Add 6 *mL* of the obtained cation resin slurry to the sample solution. Seal the sample with a lid or parafilm and place the sample on a shaker overnight. (*e*) The next day, filter the sample using a 0.2 µm polycarbonate filter and rinse the cation resin with 1–2 *mL* DD-H_2_O. (*f*) Collect the resin and recondition it in 1 *M* HNO_3_. The resin can be re-used.

*Differences between various protocols exist*, in that Goldhammer et al. [9] experienced a red discoloration of the sample when using resin prepared the previous day. Subsequently they were unable to properly precipitate Ag_3_PO_4_ in Step XIII. When preparing the cation resin within 30 min of its use they avoided this problem. They did not resolve the cause of this complication. We experienced that the samples acquired a milky white colour once the resin was added in Step XId, if the resin was prepared two days before its use (our resin was left in DD-H_2_O overnight). The whitish coloration was avoided when using the resin the same day as it was washed in DD-H_2_O. It was not possible to properly precipitate Ag_3_PO_4_ when using samples where the milky white colour had occurred. Thus, we agree with Goldhammer et al. [9]’s statement, that proper handling and rinsing of the resin before every application is crucial to the successful precipitation of Ag_3_PO_4_.

Joshi et al. [15] adjusted the pH of the dissolved MAP solution to neutral (pH 6-8) prior to the cation removal.

**Step XII. Elimination of Cl^−^**

**Fig. 6:**
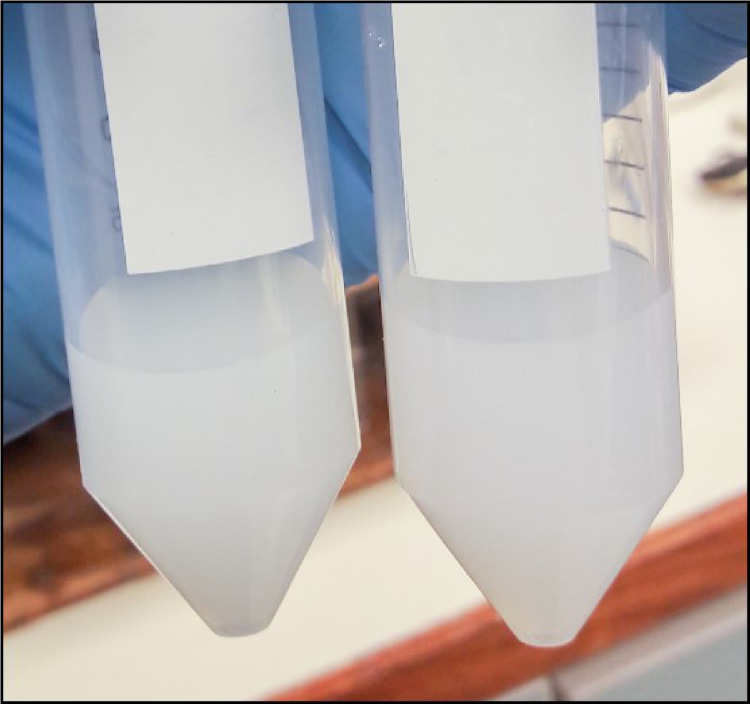
Precipitated AgCl crystals after adding AgNO_3_ to the sample solution.

Removal of Cl^−^ ions is extremely important, as Cl^−^ otherwise may react with the Ag^+^ in the final precipitation of Ag_3_PO_4_, forming AgCl [3]. Precipitation of AgCl hence both interferes with the Ag_3_PO_4_ precipitation [23] and introduces non-phosphate oxygen to the sample [3]. Chloride can be quantitatively removed by adding AgNO_3_ crystals to the sample when the pH is acidic, causing AgCl precipitation (Fig. 6) prior to the Ag_3_PO_4_ precipitation step. The low pH in the sample (<1) impede co-precipitation of Ag_3_PO_4_, and hence no P*_i_* is lost during this step. The purification step is as follows:

(q) Transfer the filtered sample solution to a small container with a small opening (e.g. 50 *mL* centrifuged tube). (*b*) Add a few AgNO_3_ crystals to the sample solution. If the sample turns whitish opaque, AgCl has precipitated (Fig. 6). (*d*) Wait at least 5 minutes and re-filter, the same filter used in Step XI can be re-used.

After this purification step, the initial freshwater sample with a volume up to 200 *L* has been reduced to about 10 *mL* of highly concentrated homogeneous P*_i_* solution ideally stripped of potential contaminants. The sample is now ready for the final Ag_3_PO_4_ precipitation.

**Step XIII Silver phosphate (Ag_3_PO_4_) precipitation**

Precipitation of insoluble silver salts, such as Ag_3_PO_4_, can be conducted by volatilization of ammonia [8]. This allows a ‘slow’ recrystallization which facilitates the growth of large and easier-to-handle Ag_3_PO_4_ crystals for oxygen isotope analysis by IRMS within a few days [8,9]. The method utilizes that Ag_3_PO_4_ precipitates from the solution at a pH around 7 (±0.5) when free Ag^+^ and P*_i_* are present. Thus the pH conditions and a high Ag^+^:P*_i_* ratio is of extreme importance to ensure complete precipitation of all P*_i_* into Ag_3_PO_4_. The ‘slow’ Ag_3_PO_4_ precipitation procedure is as follows:

(*a*) Initially add the Ag-ammine solution to the sample solution, in a Ag:P*_i_* ratio of approximately 10:1 [3]. The sample solution turns briefly white (at pH 7), and then transparent (at pH >7) once the alkaline Ag-ammine solution has been added. (*b*) Then place the sample container in an oven at 50 °*C*. Yellow Ag_3_PO_4_ crystals start to precipitate after a few hours as the amine starts to vaporize and the Ag^+^ is released [8]. Complete precipitation of the crystals may take up to two days. (*c*) After 1 to 2 days, if no yellow Ag_3_PO_4_ crystals have precipitated, check the pH of the solution. If the pH of the solution differs from pH 7 (optimal pH for Ag_3_PO_4_ precipitation conditions; [8]) adjust the pH by adding either HNO_3_ or NH_4_OH. (*d*) When crystals have formed, vacuum filter them upon a 0.2 µm polycarbonate filter and discard the supernatant. Other filters tend to ‘trap’ the Ag_3_PO_4_ crystals on their surface. (*e*) Wash the crystals extremely thoroughly with DD-H_2_O to eliminate nitrates from the previous substeps. If nitrate remains, there is an extra source of oxygen, which can interfere with the oxygen isotope analysis. (*f*) Place the filter on a Petri dish and cover it to prevent contamination and loss of crystals. Dry the filter at 50 *°C* for at least 1 day. (*g*) An extra elimination of residual organic matter might be necessary by introducing a final washing of the Ag_3_PO_4_ precipitate with hydrogen peroxide to eliminate residual organic matter by oxidation [32]. (*h*) If needed, the filter containing the Ag_3_PO_4_ crystals can be stored in a desiccator.

**Fig. 7:**
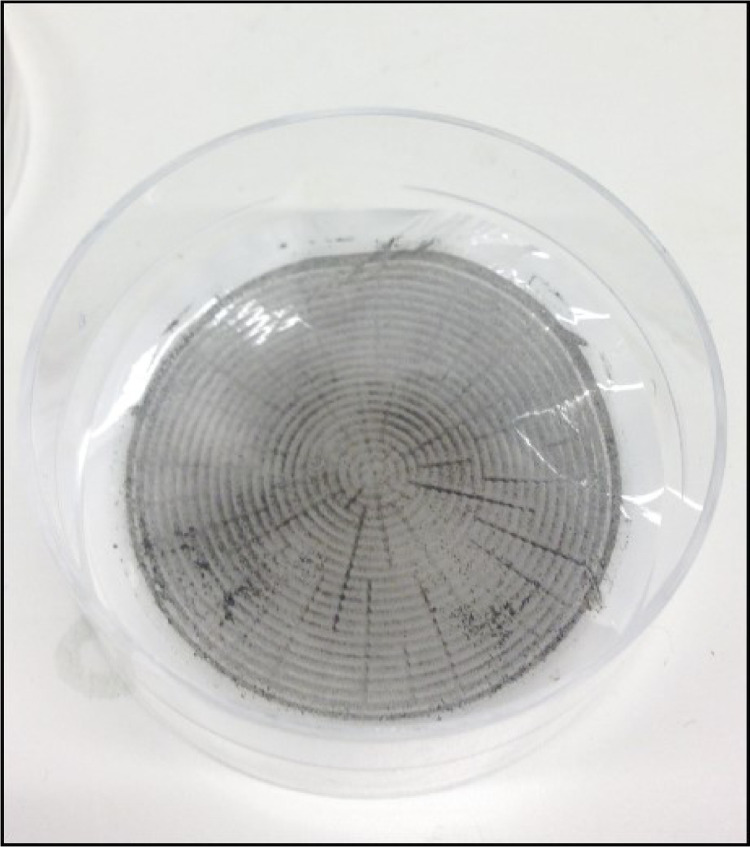
Dark colored Ag_3_PO_4_ precipitate of Step XIII.

*Notes* (*i*) It is important to repeatedly add DD-H_2_O to the solution to keep the volume as constant as possible. If left unattended (e.g. for one or several days) all the H_2_O may evaporate, which results in uncontrolled precipitation of salts. This is still fine, as the salts will be dissolved when adding DD-H_2_O, as they are mostly nitrate-based. If this happen it is vital to wash the Ag_3_PO_4_ crystals extremely well with DD-H_2_O. (*ii*) Under no circumstances should HCl or NaOH be used to adjust the pH as Cl^−^ and Na^+^ would interfere with the crystallization of Ag_3_PO_4_. (*iii*) Ag_3_PO_4_ crystals may form on the side of the tube, hence make sure to carefully detach these and transfer them to the filter as well. (*iv*) Crowson et al. [4] found that contaminated silver phosphate crystals were generally dark brown to greenish brown in colour and cohesive. We did not experience this discoloration but the crystals became dark under light (Fig. 7), probably due to photo-oxidation of the silver [23,32]; this did however not influence the analysis of the crystals.

*Differences between various protocols exist* regarding the Ag_3_PO_4_ precipitation rate: The final precipitation of Ag_3_PO_4_ can be accomplished by either a ‘slow’ [9,32] or ‘fast’ [6,23] precipitation method. Dettman et al. [6] compared the isotopic composition of the Ag_3_PO_4_ generated by the two different methods and found the resulting δ^18^O_p_ values to be within expected interlaboratory variation. Tamburini et al. [32] suggest, however, using the ‘slow’ precipitation method as an additional measure to minimize the disturbance by organic matter as suggested by Colman [3]. Yet another method is a freeze-drying ‘fast’ precipitation of Ag_3_PO_4_ suggested by Tcaci et al. [34]. This method attempts to limit the precipitation of silver oxide, being a potential source of contaminant-O in the determination of δ^18^O_p_, initiated by the addition of ammine-silver nitrate solution.

**Step XIV. Ag_3_PO_4_ crystal preparation prior to isotope ratio mass spectrometry (IRMS)**

Once the Ag_3_PO_4_ crystals have been precipitated and dried, they are prepared for isotope ratio mass spectrometry (IRMS) analysis. Such sample preparation involves:

(*a*) Transfer the Ag_3_PO_4_ crystals from the filters into silver timble capsules using a steel spatula. Weigh out ∼300 *µg* of Ag_3_PO_4_ crystals in triplicate. (*b*) After recording the Ag_3_PO_4_ weight, add a small amount (few grains) of black carbon (no need to weigh this) to each sample. (*c*) Close the capsules tight by using tweezers but absolutely do not touch them with the fingers. (*d*) Place the capsules in a 96-well micro-plate tray with holes. Once all the samples have been weighed out, seal the plate. To do this, cover the plate with parafilm, then put on the lid, and then fix the lid with tape.

*Note* There is an adverse effect from static electricity when transferring Ag_3_PO_4_ crystals to the silver thimbles using plastic spatulas. It is therefore advisable to avoid plastic spatulas and use metallic spatulas. One have to be very careful when transferring Ag_3_PO_4_ crystals to silver thimbles as this is a step where you for sure lose material.

The Ag_3_PO_4_ crystal are now ready for δ^18^O_p_ analysis.

#### Evaluation of phosphate purification and silver phosphate precipitation

One of the risks of a sequential precipitation and recrystallization process is the loss of P*_i_* in solution or of P*_i_* in a solid precipitate during processing. Losses of P*_i_*, both cumulative and for each step of the purification procedure, have been already estimated, and results can be found in Pistocchi et al. [30]. In summary, in the protocol described in Tamburini et al. [32], observed losses are not more than 2% for each purification step. However, final P*_i_* cumulative losses, calculated as the difference in P*_i_* concentration between the silver P*_i_* and the P*_i_* present in the original solution, sum up to 25% [30]. There are, indeed, losses linked to the extensive physical manipulation of the samples (e.g. removal of filters from filtering devices and scratching of crystals from the filters), which could account for the observed difference. All these losses are thus linked to abiotic processes and physical manipulation, which induce no significant isotopic fractionation. For this reason, we are confident that although not all P*_i_* was recovered in this study, there is a high level of confidence the isotopic signature of oxygen of the final purified P*_i_* is representative of the P*_i_* present in the original sample.

Oxygen contamination may also occur; this cannot be checked for until δ^18^O_p_ has been analysed, where the oxygen yield of the sample is compared to that of the pure Ag_3_PO_4_ used as standard [32]. The loss of P*_i_* and the effectiveness of the different purification steps, in producing adequately pure Ag_3_PO_4_ is difficult to evaluate during the execution. Therefore, it is important to know and pay attention to the characteristics of the precipitated crystals in each step (e.g. correct crystal colour) and evaluate whether the specific purpose of the step has been obtained (e.g. whether crystals are formed or completely dissolved).

## Final remarks

In general, the amount of added reactants and chemicals can vary from sample to sample and in many instances it depends on yield volume or quantity from the prior step. Hence, only minimum and indicative quantities are stated in the present protocol.

One of the major challenges with all the Ag_3_PO_4_ precipitation methods relates to the insufficient removal of O-bearing compounds other than P*_i_* [32] and Na^+^, Cl^−^ and multivalent cations. Thus, the purification steps are of great importance. Especially DOM is of concern as the high O content of DOM can significantly interfere with the measured fractionation of δ^18^O_p_ and persists throughout all sequential steps of the Ag_3_PO_4_ precipitation methods [25]. There is a variety of approaches to address this problem. Bearing in mind that many methods have been tested on various water matrix compositions, their effectiveness may not be reproducible for all water sample matrices. In order to achieve progress in developing and applying δ^18^O_p_ to trace P sources and P cycling in freshwater ecosystems, a better understanding of the different methods’ reliance on different water matrices is crucial. This does not only apply to the purification steps but applies for all sections presented in the present study.

In general, studies which have used δ^18^O_p_ as a tracer emphasize the importance of additional research and knowledge regarding δ^18^O_p_ data for various potential P*_i_* sources, especially for freshwater systems [7,^10^,^33^,38]. The protocol provided in the present study hopefully will contribute to enabling a broader use of δ^18^O_p_ signatures as such a tracing tool, as well as for the study of the general behaviour of P in the environment.

## Declaration of Competing Interests

The authors declare that they have no known competing financial interests or personal relationships that could have appeared to influence the work reported in this paper.

## Appendix Supplementary materials

Image, application 1

## References

[1] Blake R.E., O'Neil J.R., Garcia G.A (1997). Oxygen isotope systematics of biologically mediated reactions of phosphate: I. Microbial degradation of organophosphorus compounds. Geochim. Cosmochim. Acta.

[2] Blake R.E., O'Neil J.R., Surkov A.V (2005). Biogeochemical cycling of phosphorus: Insights from oxygen isotope effects of phosphoenzymes. Am. J. Sci..

[3] Colman A.S. (2002).

[4] Crowson R.A., Showers W.J., Wright E.K., Hoering T.C. (1991). Preparation of phosphate samples for oxygen isotope analysis. Anal. Chem..

[5] Davies C.L., Surridge B.W.J., Gooddy D.C. (2014). Phosphate oxygen isotopes within aquatic ecosystems: global data synthesis and future research priorities. Sci. Total Environ..

[6] Dettman D.L., Kohn M.J., Quade J., Ryerson F.J., Ojha T.P., Hamidullah S. (2001). Seasonal stable isotope evidence for a strong Asian monsoon. Geology.

[7] Elsbury K.E., Paytan A., Ostrom N., Kendall C., Young M., Mclaughlin K. (2009). Using oxygen isotopes of phosphate to trace phosphorus sources and cycling in lake erie. Environ. Sci. Technol..

[8] Firsching F.H. (1961). Precipitation of Silver Phosphate from Homogeneous Solution. Anal. Chem..

[9] Goldhammer T., Max T., Brunner B., Einsiedl F., Zabel M. (2011). Marine sediment pore-water profiles of phosphate d 18 O using a refined micro-extraction. Limnol. Oceanogr. Methods.

[10] Granger S.J., Heaton T.H.E., Pfahler V., Blackwell M.S.A., Yuan H., Collins A.L. (2017). The oxygen isotopic composition of phosphate in river water and its potential sources in the Upper River Taw catchment, UK. Sci. Total Environ..

[11] Gruau G., Legeas M., Riou C., Gallacier E., Martineau F., Hénin O. (2005). The oxygen isotope composition of dissolved anthropogenic phosphates: a new tool for eutrophication research?. Water Res..

[12] Hecky R.E., Kilham P. (1988). Nutrient limitation of phytoplankton in freshwater and marine environments: a review of recent evidence on the effects of enrichment1. Limnol. Oceanogr..

[13] Jaisi D.P., Blake R.E. (2014).

[14] Jaisi D.P., Kukkadapu R.K., Stout L.M., Varga T., Blake R.E. (2011). Biotic and abiotic pathways of phosphorus cycling in minerals and sediments: Insights from oxygen isotope ratios in phosphate. Environ. Sci. Technol..

[15] Joshi S.R., Li W., Bowden M., Jaisi D.P. (2018). Sources and pathways of formation of recalcitrant and residual phosphorus in an agricultural soil. Soil Syst..

[16] Karl D.M., Tien G. (1992). MAGIC: a sensitive and precise method for measuring dissolved phosphorus in aquatic environments. Limnol. Oceanogr..

[17] Kazmierczak J., Postma D., Müller S., Jessen S., Nilsson B., Czekaj J., Engesgaard P. (2020). Groundwater-controlled phosphorus release and transport from sandy aquifer into lake. Limnol. Oceanogr..

[18] Kolodny Y., Luz B., Navon O. (1983). Oxygen isotope variations in phosphate of biogenic apatites, I. Fish bone apatite-rechecking the rules of the game. Earth Planet. Sci. Lett..

[19] Li X., Wang Y., Stern J., Gu B. (2011). Isotopic evidence for the source and fate of phosphorus in Everglades wetland ecosystems. Appl. Geochem..

[20] Liang Y., Blake R.E. (2006). Oxygen isotope signature of Pi regeneration from organic compounds by phosphomonoesterases and photooxidation. Geochim. Cosmochim. Acta.

[21] Liang Y., Blake R.E. (2007). Oxygen isotope fractionation between apatite and aqueous-phase phosphate: 20-45°C. Chem. Geol..

[22] Longinelli A., Bartelloni M., Cortecci G. (1976). The isotopic cycle of oceanic phosphate, I. Earth Planet. Sci. Lett..

[23] McLaughlin K., Silva S., Kendall C., Stuart-Williams H., Paytan A. (2004). A precise method for the analysis of δ18O of dissolved inorganic phosphate in seawater. Limnol. Oceanogr..

[24] McLaughlin K., Kendall C., Silva S.R., Young M., Paytan A. (2006). Phosphate oxygen isotope ratios as a tracer for sources and cycling of phosphate in North San Francisco Bay, California. J. Geophys. Res..

[25] McLaughlin K., Paytan A., Kendalll C., Silva S. (2006). Oxygen isotopes of phosphatic compounds - Application for marine particulate matter, sediments and soils. Mar. Chem..

[26] Murphy J., Riley J.P. (1986). A modified single solution method for the determination of phosphate in natural waters. Anal. Chim. Acta.

[27] Neidhardt H., Schoeckle D., Schleinitz A., Eiche E., Berner Z., Tram P.T.K. (2018). Biogeochemical phosphorus cycling in groundwater ecosystems – Insights from South and Southeast Asian floodplain and delta aquifers. Sci. Total Environ..

[28] Nisbeth C.S., Kidmose J., Weckström K., Reitzel K., Odgaard B.V., Bennike O., Thorling L., McGowan S., Schomacker A., Kristensen D.L.J., Jessen S (2019). Dissolved inorganic geogenic phosphorus load to a groundwater-fed lake: implications of terrestrial phosphorus cycling by groundwater. Water.

[29] Paytan A., McLaughlin K., Baskaran M. (2011). Handbook of Environmental Isotope Geochemistry: Vol I.

[30] Pistocchi C., Tamburini F., Gruau G., Ferhi A., Trevisan D., Dorioz J.M. (2017). Tracing the sources and cycling of phosphorus in river sediments using oxygen isotopes: Methodological adaptations and first results from a case study in France. Water Res..

[31] Senn A.C., Kaegi R., Hug S.J., Hering J.G., Mangold S., Voegelin A. (2015). Composition and structure of Fe(III)-precipitates formed by Fe(II) oxidation in water at near-neutral pH: interdependent effects of phosphate, silicate and Ca. Geochim. Cosmochim. Acta.

[32] Tamburini F., Bernasconi S.M., Angert A., Weiner T., Frossard E. (2010). A method for the analysis of the δ18O of inorganic phosphate extracted from soils with HCl. Eur. J. Soil Sci..

[33] Tamburini F., Pfahler V., von Sperber C., Frossard E., Bernasconi S.M. (2014). Oxygen isotopes for unraveling phosphorus transformations in the soil–plant system: a review. Soil Sci. Soc. Am. J..

[34] Tcaci M., Barbecot F., Hélie J.F., Surridge B.W.J., Gooddy D.C. (2019). A new technique to determine the phosphate oxygen isotope composition of freshwater samples at low ambient phosphate concentration. Environ. Sci. Technol..

[35] Thomson-Bulldis A., Karl D. (1998). Application of a novel method for phosphorus determinations in the oligotrophic North Pacific Ocean. Limnol. Oceanogr..

[36] Vennemann W.T., Fricke C.H., Blake R.E., O'Neil J.R., Colman A (2002). Oxygen isotope analysis of phosphates : a comparison of techniques for analysis of Ag3PO4. Chem. Geol..

[37] Wetzel R.G. (2001). Limnology: Lake and River Ecosystems.

[38] Young M.B., McLaughlin K., Kendall C., Stringfellow W., Rollog M., Elsbury K.E. (2009). Characterizing the oxygen isotopic composition of phosphate sources to aquatic ecosystems. Environ. Sci. Technol..

[39] Yuan H., Li Q., Kukkadapu R.K., Liu E., Yu J., Fang H. (2019). Identifying sources and cycling of phosphorus in the sediment of a shallow freshwater lake in China using phosphate oxygen isotopes. Sci. Total Environ..

[40] Zohar I., Shaviv A., Klass T., Roberts K., Paytan A. (2010). Method for the analysis of oxygen isotopic composition of soil phosphate fractions. Environ. Sci. Technol..

