# Supplementary material for: Analysis of oxygen isotopes of inorganic phosphate (δ18Op) in freshwater: A detailed method description for obtaining oxygen isotopes of inorganic phosphate in environmental water samples
Source: MethodsX. 2022 Apr 16;9:101706. doi: 10.1016/j.mex.2022.101706 (PMC9062346; doi:10.1016/j.mex.2022.101706)
Supplement: Supplementary file 1 [file mmc1.docx]

**Appendix A - *Description of the preparation of all used chemicals and reagents***

Quantitative Pi removal by the MagIC method (Section 2.3)

- **3 *M* MgCl_2_:** The 3 *M* MgCl_2_ solution is prepared by dissolution of 610 *g* MgCl_2_∙6H_2_O (hexahydrate; MW: 203.3 *g/mol*) in deionized distilled water (DD-H_2_O) to a total volume of 1 *L*. After the salt has dissolved, filter this Mg-brine on a GF/F filter. The solution can be stored indefinitely.
- **1 *M* NaOH:** The I *M* NaOH solution is prepared by dissolution of 40 g NaOH pellets in deionized distilled water (DD-H_2_O) to a total volume of 1 *L*. The solution can be stored indefinitely.
- **1 *M* HNO_3_:** 66 *mL* of concentrated HNO_3_ is added to 934 *mL* of DD-H_2_O.

1. The solution can be stored indefinitely.

Purification and silver phosphate precipitation (Section 2.4)

- **35 *%* Ammonium nitrate:** the 35 *%* Ammonium nitrate reagent is prepared by dissolution of 538.5 *g* ammonium nitrate salt (MW = 80.052 *g/mol*) in 1000 *mL* DD- H_2_O. Stir well to dissolve the salt completely. The solution can be stored
- **5 *%* Ammonium nitrate:** the 5 *%* Ammonium nitrate reagent is prepared by dissolution of 105.5 *g* ammonium nitrate salt in 2000 *mL* DD- H_2_O. Stir well to dissolve the salt completely. The solution can be stored.
- **10 *%* NH_4_-molybdate:** the 10 *%* NH4-molybdate solution have to be prepared fresh by dissolving 53.3 *g* of ammonium molybdate salt (tetrahydrate form: 1235.86 g/mol) in 480 *mL* of DD- H_2_O (Enough for approximately 12 samples). The solution can NOT be stored.
- **Ammonium-citrate:** the Ammonium-citrate solution is prepared by adding 300 *mL* of DD- H_2_O and 140 *mL* of concentrated NH_4_OH to 10 *g* of citric acid while working under a chemical fume hood. The solution is stable at room temperature and can be stored.
- **Mg-reagent:** The Mg-reagent is prepared by dissolution of 50 *g* of MgCl_2_ (hexa-hydrate salt, MW 203.3 *g/mol*) and 100 *g* of NH_4_Cl (MW= 53.49 g/mol) in 500 *mL* DD-H_2_O. Subsequent the solution is acidified to pH 1 with concentrated HCl. The volume is then adjusted to 1 *L* with DD-H_2_O. The solution is stable indefinitely and can thus be stored.
- **1:1 and 1:20 ammonia solutions:** Measure in a volumetric cylinder concentrated NH_4_OH (50 ml for the 1:1 and 100 ml for the 1:20). Pour into an appropriate glass bottle and dilute with DD-H_2_O (50 *ml* for the 1:1 and 1900 *ml* for the 1:20). The solution can be stored.
- **0.5 *N* HNO_3_:** 33 *ml* of concentrated HNO_3_ is added to 967 *ml* of DD-H_2_O. The solution can be stored.
- **Ag-ammine:** the Ag-ammine solution is prepared by dissolving 10.2 *g* of AgNO_3_ salt (MW = 169.87 *g/mol*), and 9.6 *g* of NH_4_NO_3_ in 81.5 *ml* of DD-H_2_O. Subsequent 18.5 *ml* of concentrated NH_4_OH is added. The solution can be stored in the dark in an amber bottle.
